# Electrospun F18 Bioactive Glass/PCL—Poly (ε-caprolactone)—Membrane for Guided Tissue Regeneration

**DOI:** 10.3390/ma11030400

**Published:** 2018-03-08

**Authors:** Lucas Hidalgo Pitaluga, Marina Trevelin Souza, Edgar Dutra Zanotto, Martin Eduardo Santocildes Romero, Paul V. Hatton

**Affiliations:** 1 CeRTEV—Center for Research, Technology and Education in Vitreous Materials, Department of Materials Engineering, Federal University of São Carlos, 13565-905 São Carlos, Brazil; marina.trevelin@gmail.com (M.T.S.); dedz@ufscar.br (E.D.Z.); 2 School of Clinical Dentistry, The University of Sheffield, 19 Claremont Crescent, Sheffield S10 2TA, UK; santocildes@yahoo.com (M.E.S.R.); paul.hatton@sheffield.ac.uk (P.V.H.)

**Keywords:** membranes, guided tissue regeneration, tissue engineering, electrospinning, bone regeneration, bioactive glass

## Abstract

Barrier membranes that are used for guided tissue regeneration (GTR) therapy usually lack bioactivity and the capability to promote new bone tissue formation. However, the incorporation of an osteogenic agent into polymeric membranes seems to be the most assertive strategy to enhance their regenerative potential. Here, the manufacturing of composite electrospun membranes made of poly (ε-caprolactone) (PCL) and particles of a novel bioactive glass composition (F18) is described. The membranes were mechanically and biologically tested with tensile strength tests and tissue culture with MG-63 osteoblast-like cell line, respectively. The PCL-F18 composite membranes demonstrated no increased cytotoxicity and an enhanced osteogenic potential when compared to pure PCL membranes. Moreover, the addition of the bioactive phase increased the membrane tensile strength. These preliminary results suggested that these new membranes can be a strong candidate for small bone injuries treatment by GTR technique.

## 1. Introduction

For over 20 years, porous membranes and scaffolds have been investigated as potential biomaterials for tissue regeneration applications [1], specially in bone tissue engineering where the cells require specific structures for support and proliferation. Porous structures are able to mimic various properties of the extracellular matrix of bone tissue, giving the cells a favourable environment to grow [2]. Nowadays, it is known that this feature is a key factor to achieve a more effective bone regeneration process. Nevertheless, the ability to provide enhanced cellular support and proliferation in one structure is a challenging task, due to the need of achieving a proper balance between the material physicochemical properties and cell interaction [3,4]. To achieve this great cellular interaction, most recently developed composite membranes attempt to incorporate bioactivity into the structure in order to achieve rapid bone tissue ingrowth, mostly because the majority of biopolymers lack the ability to stimulate cellular activity [5,6,7,8].

Among the various synthetic biopolymers that are available, poly(ε-caprolactone) (PCL) is one of the most used for biomedical applications [9]. PCL has several advantages, such as an inexpensive manufacturing process, good reproducibility, easy handling, sterilization capability, non-toxicity, and biodegradability [10]. However, PCL’s cell stimulation is poor, limiting its effectiveness in supporting tissue regeneration in clinical applications. A potential way to increase its bioactivity is to incorporate well known osteoconductive and/or osteoinductive materials into a PCL matrix, such as bioactive glasses (BAG), drugs, and other components [11,12,13,14].

Bioactive glasses were first developed in the late 1960’s by Prof. Larry Hench and, since then, many compositions have been developed by various research groups for different clinical applications and to obtain specific biological outcomes [15,16,17,18]. For example, Biosilicate^®^ is a composition that, when cryslized, is applicable as a substititute of small bones parts due to its improved machinability and mechanical strenght [18]. While S53P4 has been demonstrating good results in the treatment of osteomyelitis [19,20,21]. F18 glass, on the other hand, is a new composition designed to present a greater stability (when compared to 45S5) while keeping a high level of bioactivity, being a potential alternative for hard and soft tissue regeneration [22]. This composition allows one to manufacture complex shapes, such as continuos fibers and three-dimensional (3D) pieces [19]. F18 has shown to induce vascular tissue growth, and present a broad spectrum antibacterial activity, which makes this composition highly desirable for dentistry and orthopedic applications [23,24]. A previous study demonstrated that F18 can provide antibacterial protection against a large range of bacteria (*S. aureus*, *E. coli*, *S. epidermidis,* and *P. aeruginosa*), which is a feature that can be used to enhance the biological properties of implanted biopolymers [25].

In order to enhance the regeneration process of bone tissue, it is important that scaffolds and membranes used in such applications possess a controllable and reproducible porous morphology. There are many manufacturing techniques that may be used for the fabrication of these structures [26]. However, due to its versatility and capability of producing highly porous structures, the electrospinning process presents itself as an efficient technique for the fabrication of polymeric nanofibers membranes [27]. During processing, the polymer solution and machine parameters can be adjusted to produce fibres of small diameter, shaping a structure with properties similar to that of bone extracellular matrix, which then creates an environment encouraging cellular proliferation [28]. This technique is broadly applicable for guided tissue regeneration (GTR), which is used for the treatment of periodontal disease and its associated loss of periodontal tissues and the formation of infrabony defects [29]. When applied to GTR, electrospun membranes can be used to prevent the migration of non-desirable cells into the defect site and give preference to the activity of cells that can repopulate the wound and regenerate the damaged tissue [30]. However, despite the progress of GTR therapies the adoption of these synthetic electrospun membranes remains mostly reserved to preclinical and clinical research applications, due to their lack of biointeraction [29].

The aim of this study was to incorporate F18 bioactive glass particles into an electrospun PCL matrix, thus developing a bioactive membrane that may mimic some characteristics of bone extracellular matrix (ECM) and may provide a more suitable substrate for cellular proliferation and differentiation. Since F18 bioactive glass has previously presented satisfactory results regarding degradation and bioactivity when incorporated into polymeric matrixes, such as PCL and PGS [24,31], this study focused on the characterisation of material citotoxicity and in vitro osteogenic potential of this bioactive glass when incorporated into nanostructured electrospun PCL.

## 2. Results

### 2.1. Manufacture and Characterization of Electrospun Membranes

**Figure 1:**
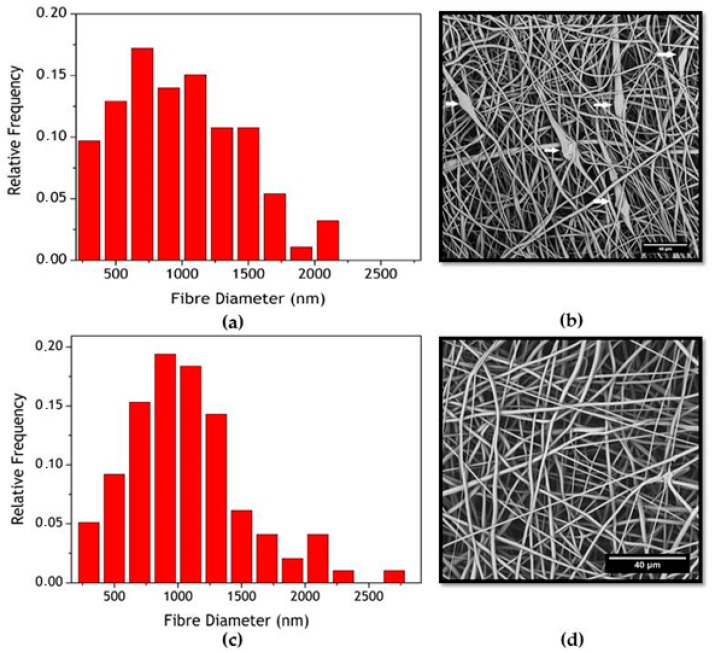
(**a**) Frequency distribution of fibres’ diameter for PCL-F18; (**b**) The correspondent SEM image of poly(ε-caprolactone) (PCL)-F18 is displayed on the right side, presenting some glass particles (white arrows); (**c**) Frequency distribution of fibres’ diameter for PCL; (**d**) The correspondent SEM image of PCL sample.

**Figure 2:**
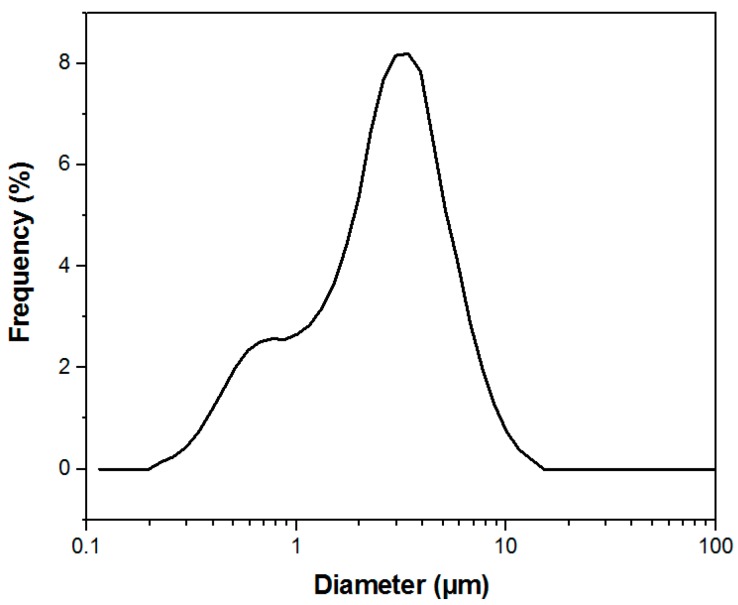
F18 bimodal particle size distribution with particles ranging from approximately 0.2 to 12 µm.

**Figure 3:**
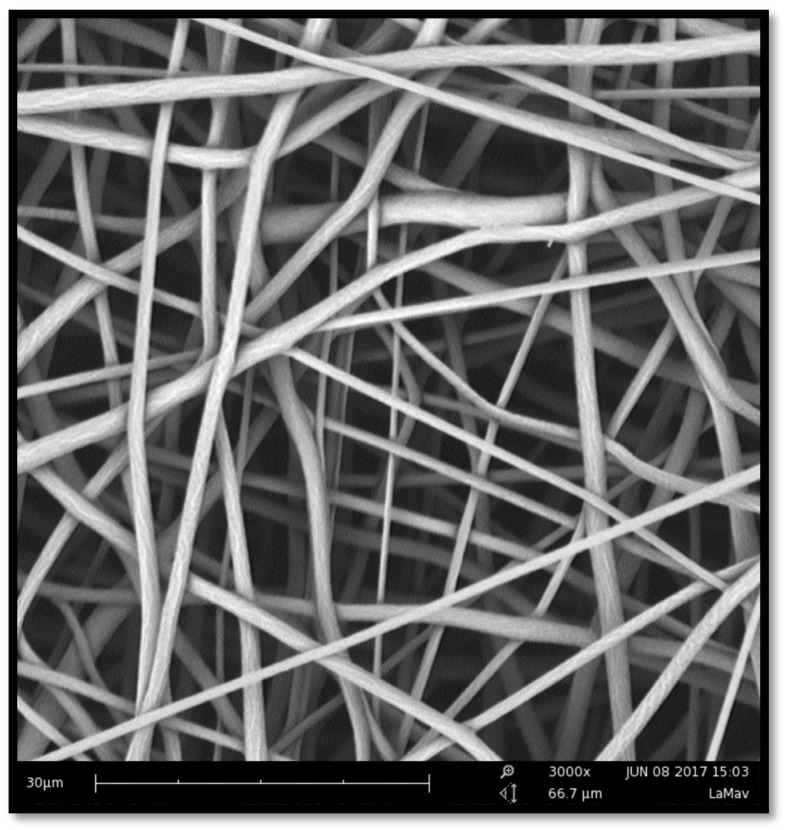
SEM image of a pure PCL membrane. The porous structure is uniform, and the fibres are randomly oriented, presenting smooth and continuous morphology. Magnification of 3000×.

**Figure 4:**
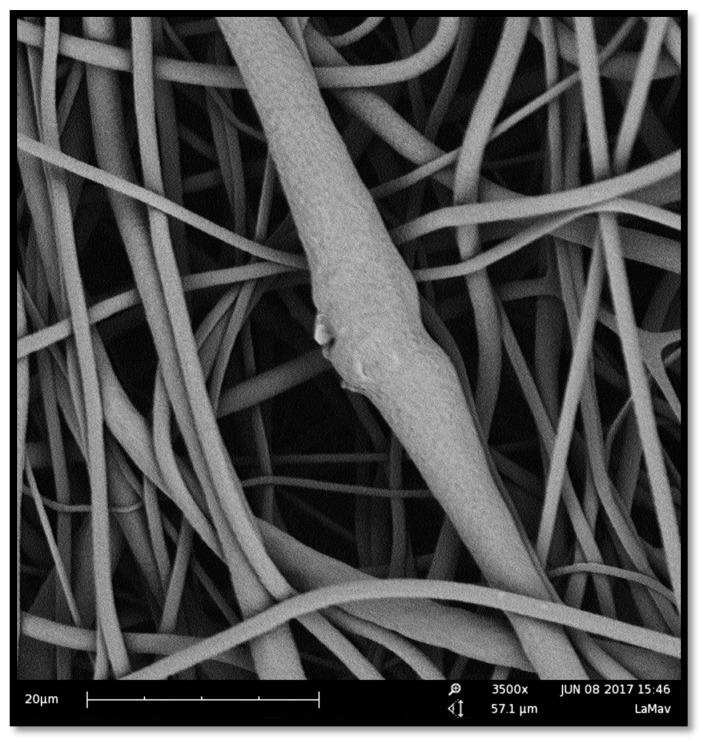
SEM image of a PCL-F18 membrane. The observed structure is slightly different than the one presented above. The fibres are randomly oriented, but beads and discontinuities of fibre are more incident. Magnification of 3500×.

The membranes were successfully manufactured using the electrospinning technique. Both compositions, pure PCL, and PCL-F18, presented a low incidence of beads and disuniformities. Moreover, the addition of glass particles affected the mean fibre diameter minimally. The size distribution was slightly altered, and diameters above 1 µm were more frequently observed in PCL-F18 membranes than in the non-composite membranes (Figure 1), this was observed since the F18 particle size distribution was bimodal, presenting particles closest to nanoscale that were possible trapped inside the PCL fibre and microscale particles that were randomly distribuited in the membrane (Figure 2). The porous surface was equally uniform on both membranes, with the glass particles located within the fibres and mostly covered by the polymer. Generally, the particles were not observed within the pore sites (Figure 3). The regions of the fibres containing the glass particles were observed to present an increased diameter due to the presence of glass inside the fibre (Figure 4). Although some average size glass particles (<20 µm) were found outside the matrix, the majority of the glass was trapped inside the fibres.

### 2.2. Cytoxicity Studies

**Figure 5:**
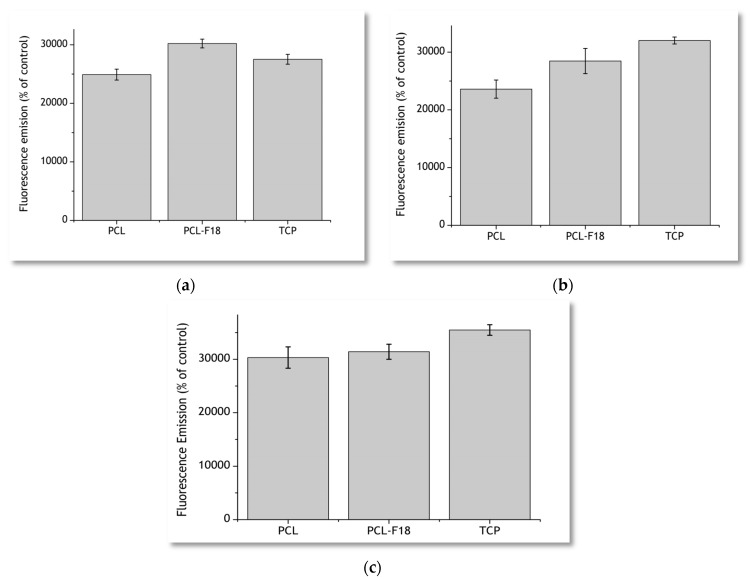
Cell viability assays performed using the PrestoBlue reagent for 3 days (**a**), 7 days (**b**) and 14 days (**c**) for pure PCL, PCL-F18, and tissue culture plastic (TCP) samples. PCL-F18 membranes presented a greater fluorescence emission than the pure and TCP in the first three days.

The results obtained via measurement of fluorescence emission of the incubated PrestoBlue solutions are presented in Figure 5. After three days of incubation, PCL-F18 membranes presented a greater fluorescence emission than the pure PCL samples and the control group of the Tissue Culture Plastic (TCP). Seven days post-seeding, part of the glass on the surface of the membrane had already been dissolved, and the difference between the flourescence emission of TCP and PCL-F18 groups inverted. However, PCL-F18 samples still presented a higher fluorescence emission than PCL alone. For these evaluation periods, no statistically significant differences between PCL-F18 and TCP were observed. After 14 days, all of the groups presented a similar trend on MG-63 cells proliferation and no statistically significant differences were observed.

### 2.3. Effect of F18 Bioactive Glass on MG-63 Cells Osteogenesis

**Figure 6:**
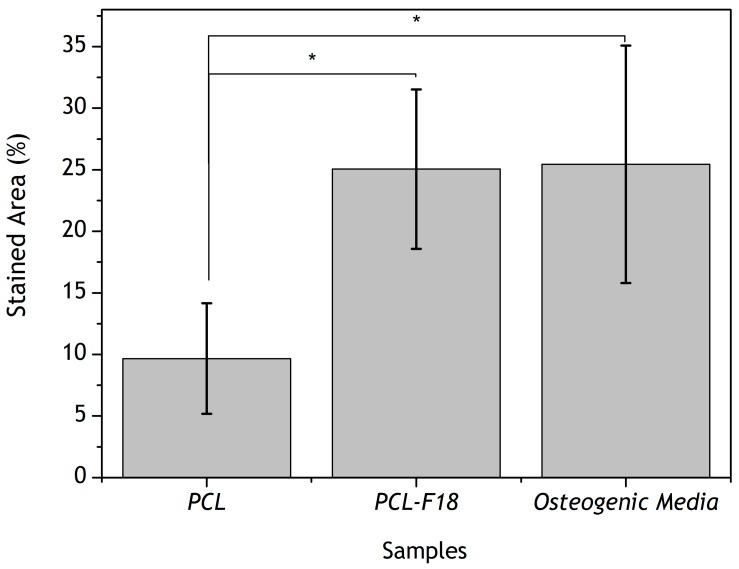
Stained area (%) of alkaline phosphatase for pure PCL, PCL-F18, and control (osteogenic medium) groups. Statistical difference is indicated by *. After 14 days, PCL-F18 samples induced a similar effect on MG-63 cells as the control osteogenic medium.

**Figure 7:**
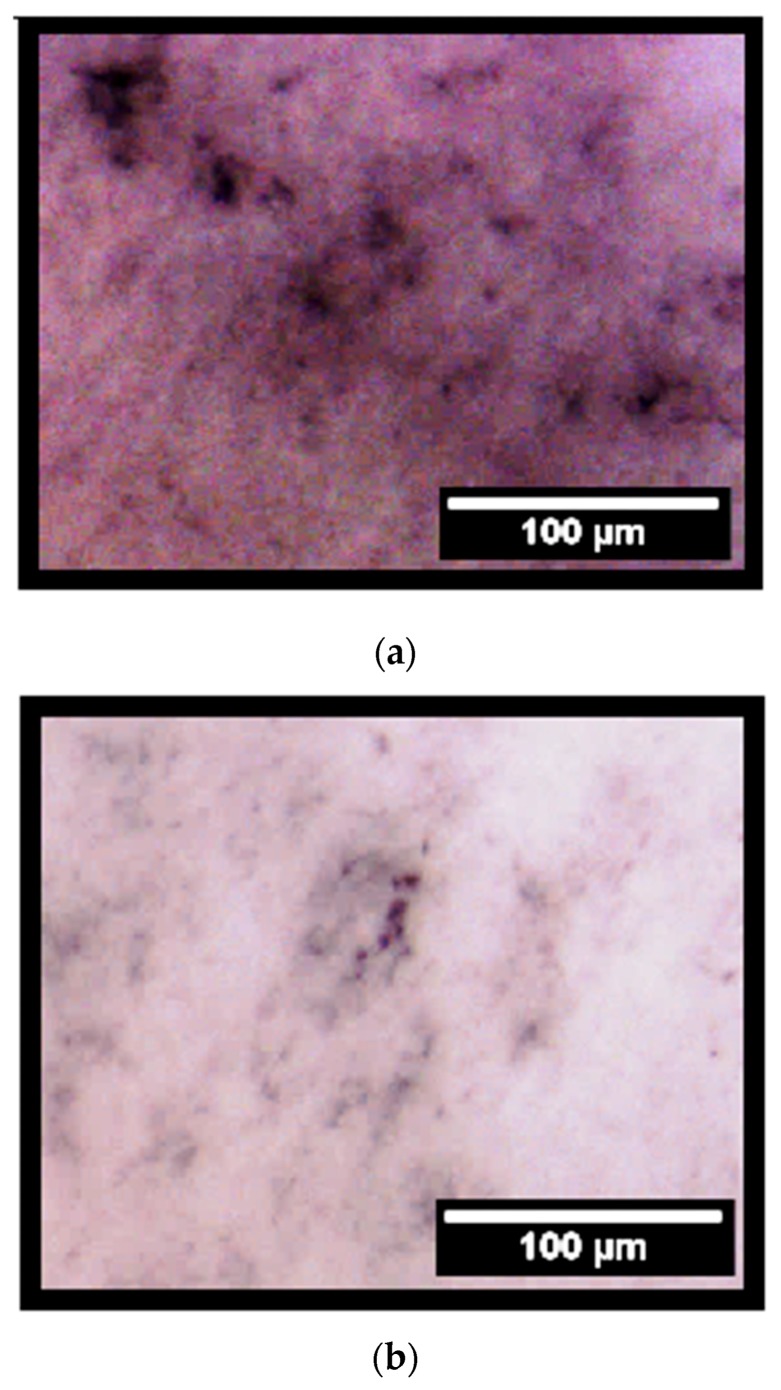

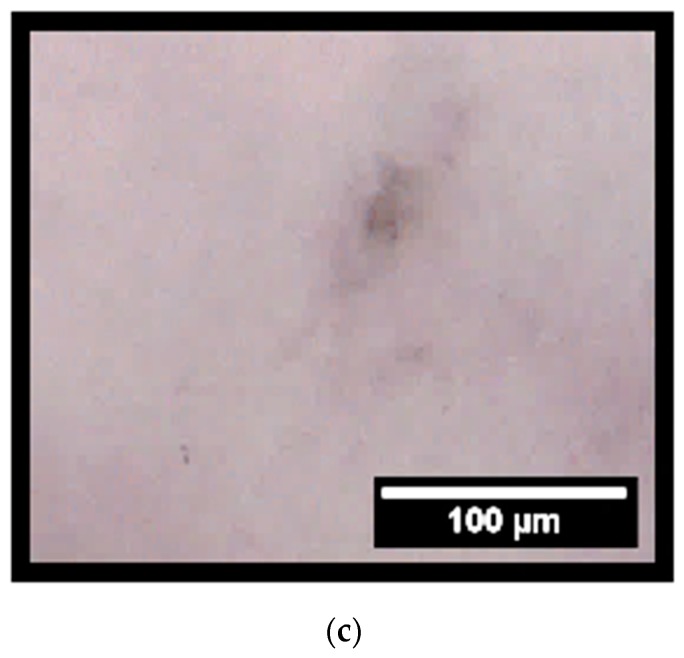
Optical micrographs of stained membranes for ALP activity. From the top to the bottom, it can be noticed the different staining levels and colour differences between PCL-F18 (**a**), PCL in osteogenic medium (**b**) and pure PCL membrane (**c**). After 14 days, PCL-F18 (**a**) samples induced a similar effect on MG-63 cells as the control osteogenic medium (**b**).

The analysis of alkaline phosphatase (ALP) activity showed that after 14 days of incubation the PCL-F18 samples induced a similar effect on MG-63 cells as the control osteogenic medium (Figure 6). Such analysis is only viable due to the reaction between alkaline phosphatase and BCIP/NBT components, resulting in an end product that is highly coloured, presenting blue to purple stains in the membranes. After treating the membranes, the stained areas can be observed on optical microscope, giving visual aspect differentiation between the cells ALP activities (Figure 7). There were no statistically significant differences between both of the groups. However, both presented a *p* ≤ 0.05 when compared to pure PCL membrane. Figure 6 depicts ALP activity for all the sample groups.

### 2.4. Mechanical Properties

The mean values for the maximum tensile strength and the maximum deformation for PCL and PCL-F18 membranes, as well as their standard deviations, are presented in Table 1. The tensile tests demonstrated that the membranes that are incorporated with F18 biactive glass are approximatelly 31% more resistant to tensile load than the pure PCL membranes. However, the strain at break data presented values that suggested a greater plasticity of pure PCL samples. Statistical analyses showed *p*-value < 0.05 for this data.

## 3. Discussion

Periodontal osseous surgery aimed at tissue repair normally leads to unsatisfactory and unpredictable results due to the the ingrowth of connective tissue into the defect, compromising the complete healing of bone [30]. In order to overcome this problem, barrier membranes are frequently implanted to prevent the access of surrounding soft tissues to the defect. However, the barrier membranes currently available generally lack bioactivity and cannot encourage bone regeneration besides that induced by the mechanical separation. Several studies show that the application of nanostructured porous membranes that are doped with a bioactive phase may be a suitable strategy for guided tissue regeneration (GTR) procedures [4,6,7,8,32]. In this study, the manufacture of polymeric membranes with bioactive properties was sucessfully done using the electrospinning technique. The uniformity and fiber size range observed in this study were also reported by similar studies, indicating that nano to micron scale fibers can be efficient for guided bone regeneration, as these structures can mimic the host tissue pore size, hence promoting new tissue growth [28]. The incorporation of the microsized glass particles slightly increased fibre diameter, as expected, up to a maximum of 3 µm. Although an increased fibre diameter may be associated with less effective regeneration, the diameters observed in this study were acceptable for GTR membranes. This is not the first time that such result was obtained, as Ren et al. [33] also observed that the presence of BAG microparticles might increase the fibres diameter variance by creating some large sized fibres during the spinning process.

Regarding the results that were observed in the cytotoxicity assay, the PCL-F18 membranes increased the metabolic activity of MG-63 osteoblast-like cells when compared to those cultured on pure PCL membranes and TCP (control group), especially during the initial evaluation periods. This rapid response may be attributed to the rapid dissolution of the F18 glass particles on the surface of the fibers, releasing ions, such as Ca, Na, Si, and P to the medium. This ion leaching phenomenon was evaluated for F18 in several other studies [19,20,21,34] and is a well documented process for bioactive glasses in the literature [15,16,17,18]. These ionic products of bioactive glass dissolution are linked to an enhanced stimulation of cell proliferation. Xynos et al. [35] showed that bioactive glasses can induce mitogenic stimulation and can increase the proliferation of osteoblast cells due to an increase in the concentration Si ions in the medium. After seven days, cell cultured on the PCL-F18 composite membranes presented a greater metabolic activity than pure PCL membranes, although not significantly different to that from TCP. This may be linked to a lower availability of glass particles, since the easily accessible particles had already been consumed. For new particles to become available, the polymer would have to degrade first. This trend was also reported by other studies that incorporated a bioactive phase into a synthetic biopolymer matrix [6,10]. The enhancement of the osteogenic potential of the membranes may be linked to the incoporation of F18 glass, as this bioactive phase presents demonstrated osteogenic and osteoconductive properties that are highly desired for membrane for GTR proposes [4,36]. This glass composition has proven to be effective for faster proliferation of fibroblasts and osteoblasts cells in in vitro and in vivo tests [23,24]. Our study presented a similar trend using MG-63 cells. As F18 particles dissolved, a microenvironment that improved cellular activity and function was created. However, considering that PrestoBlue does not give an information about the quantity of cells, it may only be suggested that the duration of this stimulation was limited to the initial evaluation period, which is mainly due to the rapid glass dissolution. Santocildes et al. [6] and Fabbri et al. [37] reported similar results when incoporating different bioactive glasses into PCL membranes. Both of the authors stated that the composite membranes generally presented good in vitro biocompatibility, and also observed that cell proliferation was lower for the composites and significantly lower for pure PCL when compared to the tissue culture plastic, likely due to the low wettability of PCL. Other authors also studied how the glass particles altered the degradation of PCL in vitro. Tamjid et al. [38] suggests that BAG particles may increase not only the wettability of PCL, but also its surface roughness, aiding the polymer corrosion process. The same trend was detected by Poh et al. [39], evaluating the polymer behaviour under accelerated degradation conditions.

The alkaline phosphatase (ALP) activity was assessed to determine the osteogenic potential of PCL-F18 composite membranes. For this purpose, groups of membranes were cultured in osteogenic-supplemented (control and pure PCL groups) or non-osteogenic-supplemented cell culture media (PCL-F18 and pure PCL groups). ALP analysis indicated that the PCL-F18 membranes could induce a similar amount of mineralization than the control osteogenic-supplemented medium. Several studies also suggested a similar trend when incorporating bioactive glasses into polymer membranes. According to Leal et al. [5] and several other authors, BAG incorporation into a biopolymer matrix can significantly enhance mineralization and cellular activity, stimulating ALP activity of different cell lines. This may be used to grant potential osteoconductivity and/or osteoinductivity properties to these materials for GTR and GBR (Guided Bone Regeneration) applications [5,32,40,41]. Besides, these bioactive composites materials have also shown to have improved mechanical properties when compared to other polymeric membranes for GTR, which was also observed in this study [42].

The mechanical tests revealed that the PCL-F18 composite membranes presented a superior average maximum tensile strength than the pure PCL membranes. This increase may be related to the filler effect created by the addition of a ceramic phase, where the glass particles hinder the movement of the polymer chain and reduce the amount of readily extendable material in the matrix [43]. Nevertheless, the plastic deformation was slightly affected by the glass particles, as expected in composites in which a polymer matrix is reinforced with a rigid ceramic phase. In this case, an increase in the modulus and/or strength usually occurs at the expense of elongation at break [43]. In this study, the F18 particle-size distribution was quite wide and the smaller particles were incorporated into the PCL fibers, probably increasing the membranes’ mechanical resistance. Whereas, the average size particles were observed in both inside and outside the membrane’s matrix, probably interfering on the composites’ mechanical resistance. Overall, the difference between the groups (pure PCL and PCL + F18) was not statistically significant. According to other studies presented by Li et al. [44] and Bottino et al. [41], a composite membrane that presents a tensile strength around 2 to 3 MPa has already a potential good resistance to meet the mechanical requirements of GTR applications. In this study the PCL-F18 membranes presented a mean value of approximately 5 MPa, which suggested that this novel composite could be a potentially viable alternative for periodontal osseous surgery.

## 4. Materials and Methods

### 4.1. Bioactive Glass Manufacturing

F18 is a bioactive glass composition that belongs to the glass system SiO_2_-CaO-Na_2_O-MgO-K_2_O-P_2_O_5_-B_2_O_3_ [24]. The glass preparation is described in detail elsewhere [13,14]. Briefly, analytical grade chemicals were mixed and melted in a platinum crucible at 1350 °C for 3 h. The bioactive glass powder was then produced by milling and sieving the glass frits obtained after quenching the melt in distilled water. A mean particle size of approximately 5 μm was used (particle size distribution was verified by laser scattering in the equipment Horiba (LA-930, Kyoto, Japan).

### 4.2. Glass/Polymer Solution Preparation

Poly (ε-caprolactone (PCL, Sigma Aldrich, Irvine, UK) with an average M_w_ = 80,000 was dissolved in a blend of dichloromethane (DCM) and dimethylformamide (DMF) (volume ratio of 90:10). For the fabrication of pure PCL membranes, the control group, a spinning solution of concentration 10 wt %. of PCL was used. For the fabrication of F18-PCL membranes, a weight ratio of 10:1 PCL:F18 glass powder was used. The glass powders were added to the polymer solution under continuous stirring for three hours to attain homogenization.

### 4.3. Electrospinning Process

The polymer/glass solution was inserted into a 5-mL plastic syringe and pumped at a feed rate of 3 mL/h through a 17 kV electric field generated by an Alpha IV Bradenburg (Bradenburg, UK) power source. The fibres were then collected on a collector covered with aluminum foil and located at a distance of 18 cm from the tip of the metallic needle. The membranes were separated from the foil to be prepared for the in vitro tests.

### 4.4. Cell Viability

The metabolic activity of MG-63 osteoblast-like cells was measured using the PrestoBlue reagent (Resazurin-based dye; Thermo Fisher, Waltham, MA, USA) after seeding and incubation. The total number of membranes tested was 8 (*n* = 4, PCL and PCL-F18) with the pure PCL membranes used as control for cell viability, and tissue culture plastic (TCP) used as control for cell proliferation. The concentration of cells at seeding was 40.000 cells per sample, and measurements of metabolic activity were obtained after 3, 7, and 14 days of incubation utilizing a microplate fluorescence reader (FLx 800 Bio-Tek Instruments, Swindon, UK). The evaluation was executed using an excitation wavelength of 540 nm and an emission wavelength of 635 nm.

Prior to the fluorescence analyses, the cell culture media was removed, the cells were rinsed with sterile PBS, and a solution of 10 vol % PrestoBlue (Thermo Fisher, Waltham, MA, USA) in cell culture medium was added (700 µL per well). After 90 min of incubation, the fluorescence analyses were performed by taking three samples of 200 µL each from each well and placed in a 96-well plate. The PrestoBlue solution was finally removed from the 24-wells plate and replaced with fresh cell culture medium to continue the monitoring of the culture.

### 4.5. Alkaline Phophatase Assay

For the measurement of alkaline phosphatase activity, one BCIP/NBT tablet (SigmaFastTM BCIP-NBT; Sigma Aldrich, St. Louis, MO, USA) was dissolved in 10 ml of distilled water and covered for the protection from light. A washing buffer was prepared by adding 0.05% Tween 20 to Dulbecco’s PBS without Ca++ and Mg++ (Thermo Fisher, Waltham, MA, USA)).

The MG-63 osteoblast-like cells were washed with PBS after the removal of the medium, and the membranes were covered with neutral buffered formalin (10%) for fixation. Afterwards, the membranes were washed using the washing buffer, and a volume of the BCIP/NBT substrate solution was added to fully cover the membranes. The plates were then incubated at room temperature in the dark for 10 min, the membranes were washed again using the washing buffer, and PBS was added to each well.

The presence of alkaline phosphatase activity on the membranes was detected by a change of colour to dark purple, which was analyzed through image analysis. For this, four micrographs were taken from each membrane. The membranes were divided in four quadrants and the area of the stained regions was measured using ImageJ 1.48 software (NIH, Bethesda, MD, USA).

### 4.6. Scanning Electronic Microscope (SEM) Imaging

SEM images were taken using two different microscopes (Jeol JSM6400, Tokyo, Japan) and Phenom Pro, (PhenomWorld, Eindhoven, The Netherlands). The Jeol microscope was used for fibre morphology observation, and the Phenom Pro microscope was used for fibre diameter measurement and glass particles detection. All the samples were previously prepared employing gold sputter coating and carbon conductive tape to guarantee proper adhesion and electronic conductivity.

### 4.7. Fibre Diameter Measurement

After SEM images acquisition, 10 images of each sample were selected and 10 random fibres from each image were measured using ImageJ 1.48 software (NIH, Bethesda, MD, USA). At least 100 points were collected for each composition.

### 4.8. Mechanical Tests

The membranes were mechanically tested in a MTS Universal Testing Machine (Criterion Model 43, Eden Prairie, MN, USA), equipped with a 1kN load cell, at a test speed set at 10 mm/min. The samples were cut into small strips of dimensions 40 mm × 5 mm. Stress-strain curves were obtained and processed using MTS TestSuite TW software (MTS, Eden Prairie, MN, USA). The ultimate tensile strength (UTS) was considered as the maximum tension extracted from the same stress-strain plots. Six samples of each composition were tested, and the data analysis was performed using Origin 8.0 software (OriginLab Corporation, Northampton, MA, USA).

### 4.9. Statistical Analysis

Statistical analyses were performed on OriginPro 8.5 (OriginLab Corporation, Northampton, MA, USA) software using one-way ANOVA, in order to determine significance. In all cases, *p* values ≤ 0.05 were considered to be statistically significant. To probe normal distribution hypothesis, a residual analysis was conducted using Minitab 17 software (Minitab Inc., State College, PA, USA).

## 5. Conclusions

Electrospun membranes incorporated with a highly bioactive glass (F18) were successfully manufactured and were tested for mechanical and biological properties. The in vitro tests suggested the successful development of a biocompatible material exhibiting a greater osteogenic potential than PCL alone. The mechanical characterization of the membranes revealed a suitable tensile strength and toughness for GTR applications. This preliminary study has demonstrated promising results for the application of this novel bioactive composite as a barrier to prevent connective tissue migration into bony defect sites and to promote the growth of progenitor bone in periodontal osseous surgery.

## Figures and Tables

**Table 1:** Mean elongation (%) and tensile strength for PCL and PCL-F18 membranes.

Sample	Tensile Strength (MPa)	Elongation (%)
PCL	3.7 ± 0.5	230.0 ± 39.1
PCL-F18	4.8 ± 0.9	170.4 ± 34.5
